# Metagenome Sequencing of Prokaryotic Microbiota from Two Hypersaline Soils of the Odiel Salt Marshes in Huelva, Southwestern Spain

**DOI:** 10.1128/genomeA.00140-18

**Published:** 2018-03-01

**Authors:** Blanca Vera-Gargallo, Laura Navarro-Sampedro, Modesto Carballo, Antonio Ventosa

**Affiliations:** a Department of Microbiology and Parasitology, Faculty of Pharmacy, University of Sevilla, Seville, Spain; b Servicio de Biología, Centro de Investigación, Tecnología e Innovación (CITIUS), University of Sevilla, Seville, Spain

## Abstract

Two 454 shotgun metagenomes were sequenced from hypersaline soil samples collected in the Odiel salt marsh area in Huelva, southwestern Spain. Analysis of contigs and 16S rRNA-related sequences showed that Halobacteria, Balneolaeota, and Bacteroidetes were the dominant groups. Rhodothermaeota and Nanohaloarchaeota were also abundant.

## GENOME ANNOUNCEMENT

Hypersaline environments are extreme habitats characterized by high salt concentrations, among other stressors. These environments mainly comprise aquatic (such as lakes, salterns, and deep hypersaline anoxic basins) and terrestrial habitats (sediments and soils) (1). Extensive studies have been performed to investigate the microbiota dwelling in hypersaline aquatic habitats (2, 3). However, only limited and partial data are available regarding the prokaryotic community inhabiting saline soils (4). The Odiel salt marshes are situated at the estuary of the Odiel and Tinto rivers. The area has been found to be affected by the metal load transported by the Tinto and Odiel rivers, which drain the Iberian Pyrite Belt (5), as well as by industrial effluents from the chemical pole located in Huelva (6).

The objective of this study was to assess the prokaryotic community of saline soils from the Odiel salt marshes and evaluate its variation from one year to the next. The sampling site (37.207218 N, −6.965999 W) was located in a high marsh area that was devoid of plants and for which rain events are the main water inputs, with flooding occurring only in case of very high tides. Conductivities of 24 and 55 dS/m were determined in 1:5 water extracts for the samples retrieved in October 2013 (designated SMO1) and November 2014 (SMO2). pH values measured in 1:2.5 extracts of SMO1 and SMO2 were 7.8 and 8.9, respectively.

Total DNA was extracted from 10 g of each soil sample using the FastDNA spin kit for soil (MP Biomedicals) according to the manufacturer’s instructions. Further cleaning of DNA was performed by the phenol-chloroform method as described in Green and Sambrook (7). Sequencing was carried out with a Roche Sequencer FLX (GS FLX), and 1,289,630 and 839,941 reads were obtained for SMO1 and SMO2, respectively. Average read lengths were 628 bp for SMO1 and 629 bp for SMO2. Metagenomic reads were coassembled *de novo* using Newbler v2.9 (8) and assembly assessed with QUAST v.2.3 (9), resulting in a total of 25,001 contigs longer than 1 kb with an *N*_50_ contig size of 1,857 bp. Contigs were annotated using MEGAN v6.5.10 lowest common ancestor (LCA) algorithm (10), and extraction, refinement, and quality assessment of metagenome assembled genomes (MAGs) were accomplished with MetaBAT v0.26.3 (11), VizBin v0.9 (12), and CheckM v1.0.5. Additionally, BLASTn searches of metagenomic reads of SMO1 and SMO2 against RDP database v11.4 (13) were performed to retrieve and classify 16S-related rRNA reads.

Taxonomic assignment of contigs revealed that the microbial community comprised 51.2% Euryarchaeota, 21.3% Balneolaeota, and 16.1% Bacteroidetes. Rhodothermaeota (2.7%), Nanohaloarchaeota (1.5%), Cyanobacteria (1.3%), Gammaproteobacteria (1.2%), and Gemmatimonadetes (1.1%) also constituted more than 1% of the community. The most abundant genera were related to Halobacteria (Haloarcula, Halorubrum, Halolamina, and Salinigranum), Balneolaeota (Fodinibius and Gracilimonas), Bacteroidetes (Salinimicrobium), and some members of Proteobacteria (Halomonas and Marinobacter).

The metagenomic data obtained will be of great value in the study of halophiles and the microbiota of hypersaline environments.

### Accession number(s).

Metagenomic data sets obtained in this study have been deposited in DDBJ/ENA/GenBank under the BioProject number PRJNA318875. Accession numbers of raw reads available at the NCBI Sequence Read Archive are SRR5753724 and SRR5753725.

## References

[1] Ventosa A, Mellado E, Sánchez-Porro C, Márquez MC 2008 Halophilic and halotolerant micro-organisms from soils, p 87–115. *In* Dion P, Nautiyal CS (ed), Microbiology of extreme soils, 2nd ed Springer, Berlin, Germany.

[2] Ventosa A, Fernández AB, León MJ, Sánchez-Porro C, Rodriguez-Valera F 2014 The Santa Pola saltern as a model for studying the microbiota of hypersaline environments. Extremophiles 18:811–824. doi:10.1007/s00792-014-0681-6. 25129545

[3] Oren A 2015 Halophilic microbial communities and their environments. Curr Opin Biotechnol 33:119–124. doi:10.1016/j.copbio.2015.02.005. 25727188

[4] Xie K, Deng Y, Zhang S, Zhang W, Liu J, Xie Y, Zhang X, Huang H 2017 Prokaryotic community distribution along an ecological gradient of salinity in surface and subsurface saline soils. Sci Rep 7:1–10. doi:10.1038/s41598-017-13608-5. 29042583 PMC5645410

[5] Hierro A, Olías M, Ketterer ME, Vaca F, Borrego J, Cánovas CR, Bolivar JP 2014 Geochemical behavior of metals and metalloids in an estuary affected by acid mine drainage (AMD). Environ Sci Pollut Res Int 21:2611–2627. doi:10.1007/s11356-013-2189-5. 24096526

[6] Bolívar JP, García-Tenorio R, Mas JL, Vaca F 2002 Radioactive impact in sediments from an estuarine system affected by industrial wastes releases. Environ Int 27:639–645. doi:10.1016/S0160-4120(01)00123-4. 11934113

[7] Green MR, Sambrook J 2017 Isolation of high-molecular-weight DNA using organic solvents. Cold Spring Harb Protoc 2017:pdb.prot093450. doi:10.1101/pdb.prot093450. 28373491

[8] Margulies M, Egholm M, Altman WE, Attiya S, Bader JS, Bemben LA, Berka J, Braverman MS, Chen Y-J, Chen Z, Dewell SB, de Winter A, Drake J, Du L, Fierro JM, Forte R, Gomes XV, Godwin BC, He W, Helgesen S, Ho CH, Hutchison SK, Irzyk GP, Jando SC, Alenquer MLI, Jarvie TP, Jirage KB, Kim J-B, Knight JR, Lanza JR, Leamon JH, Lee WL, Lefkowitz SM, Lei M, Li J, Lohman KL, Lu H, Makhijani VB, McDade KE, McKenna MP, Myers EW, Nickerson E, Nobile JR, Plant R, Puc BP, Reifler M, Ronan MT, Roth GT, Sarkis GJ, Simons JF, Simpson JW, Srinivasan M, Tartaro KR, Tomasz A, Vogt KA, Volkmer GA, Wang SH, Wang Y, Weiner MP, Willoughby DA, Yu P, Begley RF, Rothberg JM 2006 Genome sequencing in microfabricated high-density picolitre reactors. Nature 441:120–120. doi:10.1038/nature03959. PMC1464427 16056220

[9] Gurevich A, Saveliev V, Vyahhi N, Tesler G 2013 QUAST: quality assessment tool for genome assemblies. Bioinformatics 29:1072–1075. doi:10.1093/bioinformatics/btt086. 23422339 PMC3624806

[10] Huson D, Auch A, Qi J, Schuster S 2007 MEGAN analysis of metagenome data. Genome Res 17:377–386. doi:10.1101/gr.5969107. 17255551 PMC1800929

[11] Kang DD, Froula J, Egan R, Wang Z 2015 MetaBAT, an efficient tool for accurately reconstructing single genomes from complex microbial communities. PeerJ 3:e1165. doi:10.7717/peerj.1165. 26336640 PMC4556158

[12] Laczny CC, Sternal T, Plugaru V, Gawron P, Atashpendar A, Margossian H, Coronado S, der Maaten L, Vlassis N, Wilmes P 2015 VizBin - an application for reference-independent visualization and human-augmented binning of metagenomic data. Microbiome 3:1. doi:10.1186/s40168-014-0066-1. 25621171 PMC4305225

[13] Cole JR, Wang Q, Fish JA, Chai B, McGarrell DM, Sun Y, Brown CT, Porras-Alfaro A, Kuske CR, Tiedje JM 2014 Ribosomal Database Project: data and tools for high throughput rRNA analysis. Nucleic Acids Res 42:D633–D642. doi:10.1093/nar/gkt1244. 24288368 PMC3965039

